# Lived experience of depression in women living with Human Immunodeficiency Virus in Gondar City health facilities, Northwest, Ethiopia: A phenomenological study

**DOI:** 10.1371/journal.pone.0352678

**Published:** 2026-06-29

**Authors:** Tadele Amare Zeleke, Tadesse Awoke Ayele, Zewditu Abdissa Denu, Lillian Mwanri, Telake Azale

**Affiliations:** 1 Department of Psychiatry, College of Medicine and Health Science, University of Gondar, Gondar, Ethiopia; 2 Department of Epidemiology and Biostatistics, Institute of Public Health, College of Medicine and Health Sciences, University of Gondar, Gondar, Ethiopia; 3 Department of Anesthesia, College of Medicine and Health Sciences, University of Gondar, Gondar, Ethiopia; 4 Research Centre for Public Health, Equity and Human Flourishing, Torrens University Australia, Adelaide Campus, Adelaide, South Australia; 5 Department of Health Promotion and Behavioral Sciences, Institute of Public Health, College of Medicine and Health Sciences, University of Gondar, Gondar, Ethiopia; Injibara University, ETHIOPIA

## Abstract

**Background:**

Many women living with HIV (WLHIV) in worldwide experience mental health conditions, particularly depression, which can negatively affect their overall wellbeing. Their lived experiences play a crucial WLHIV has been explored in various global context, there is limited research focusing on the specific women’s lived experience of depression in Ethiopia. Understanding these experience is essential to inform the development of culturally appropriate and targeted mental health interventions and support system.

**Method:**

A qualitative phenomenological design was employed to explore women’s lived experience of depression in Ethiopia. Criterion-based sampling was used, and recruitment continued until data saturation was achieved. Data were collected through in-depth face to face interviews with 16 participants attending health facilities. An inductive approach was used for analysis, with code derived from the data using Ritchie and Spencer’s analytical framework. Subsequently, Interpretive Phenomenological Analysis was applied to identify key themes and subthemes. Data analysis was supported by MAXQDA (version 22) software.

**Results:**

This study identified key themes on women’s lived experience of depression, including symptoms, meaning, perceived causes, perpetuating and relieving factors, treatment perceptions, coping strategies, and challenges. Depression affected emotional, physical and social well-being, and was shaped by factors such as HIV status, stigma, financial hardship, and lack of support. Women used diverse coping strategies, including spiritual practices, social support, and daily routines. Some preferred spiritual healing over professional care. Depression also negatively affected memory, motivation, self-care, and ART adherence, contributing to isolation, unemployment, and decline health.

**Conclusion:**

Depression among WLHIV shaped by intersecting social, economic, and health related challenge. Addressing this requires the Ministry of Health to implement integrated, culturally sensitive mental health interventions and strengthen mental health awareness, combining psychosocial support, spiritual care, and improved access to professional services.

## Introduction

The World Health Organization (WHO) reported in 2025 that Human Immunodeficiency Virus (HIV) remains a major global public health challenge, with approximately 40.8 million people (37.0–45.6 million) living with HIV worldwide [1]. Nearly two-thirds of these infections occur in the African region, reflecting persistent inequalities in prevention, diagnosis, and treatment access [2]. Women are disproportionately affected by the epidemic, particularly in sub-Saharan Africa, where discrimination, insecurity, gender-based violence, and poverty continue to expose women and girls to heightened HIV risk. Globally, women accounted for approximately 59% of new adult HIV infections in 2018 [3].

Violence against women increases HIV vulnerability by causing physical harm, such as genital injuries, and limiting women’s ability to negotiate safe sex [4]. It also creates barriers to HIV testing and treatment, as fear of abuse, stigma, and social consequences may prevent women from seeking care [4,5]. Additionally, emotional trauma and gender inequality reduce treatment adherence, access to services and social support, further worsening health outcomes [5–7].

The emotional toll of living with both HIV and violence is profound and enduring. Women facing these intersecting challenges commonly experience depression, anxiety, post-traumatic stress disorder (PTSD), and chronic emotional distress [8]. For women living with HIV, exposure to violence deepens feelings of fear, shame, guilt, and isolation. Many conceal both their HIV status and experiences of abuse to protect themselves from further harm, but this secrecy often results in loss of social support and increased emotional strain [6,9]. Women who experience violence are significantly more likely to suffer from depression and anxiety than those who have not been abused [8]. The double stigma of HIV and violence often forces women into silence, increasing isolation and worsening psychological distress [6].

By 2021, women accounted for approximately 63% of all new HIV infections in sub-Saharan Africa [10]. An HIV diagnosis is frequently accompanied by shock, fear, and extreme psychological burden for women [11]. Social support from family members, peers, community organizations, and government services plays a critical role in enabling women to cope with HIV and manage their condition as normally as possible [12].

Despite the importance of support, disclosure of HIV status often results in severe social consequences. More than half of women who disclose their HIV status report rejection by husbands or family members, and some are prohibited from visiting their children [13]. Social stigma involves labeling individuals based on socially significant characteristics, linking these labels to negative stereotypes, and subjecting individuals to discrimination, separation, and loss of social status [14,15]. HIV-related stigma discourages many women from seeking medical care and psychosocial support. Stigma manifests in multiple forms, including self-stigma, social stigma, and stigma within healthcare settings [16]. Gender strongly shapes how stigma is experienced by women living with HIV [17]. Social stigma from family members, communities, and healthcare providers can discourage care-seeking [17], while self-stigma characterized by internalized shame, guilt, and fear further limits disclosure and access to services [18]. In many developing countries, entrenched gender norms intensify these stigmas, reducing social support and worsening mental health outcomes [17]. Some women perceive infection control measures taken by healthcare providers as discriminatory, reinforcing feelings of marginalization and mistrust [16]. In addition, HIV-positive mothers face unique socioeconomic challenge. Opportunistic infections may limit breastfeeding, necessitating costly alternatives. Combined with medical expenses and caregiving responsibilities, these challenge place substantial economic strain on women and heighten their cultural and socioeconomic vulnerability [19].

Evidence from diverse settings highlights the widespread impact of stigma and psychological distress among women living with HIV. In Saudi Arabia, people living with HIV(PLWHIV) often avoid disclosing their status due to fear of discrimination, stigma, and shame, frequently relying on spiritual coping strategies to manage fears of illness, death, and social isolation [20]. Women living with HIV commonly experience unstable marriages and child-related stigma, leading to nondisclosure within families and communities [21].

In India, qualitative studies have reported lack of social support, high levels of stigma and discrimination, reduced quality of life, mental health problems, and negative coping strategies among individuals diagnosed with HIV [22]. Women often report emotions such as sadness, fear, despair, and pain following diagnosis feelings recognized as precursors to depression [23]. Among African American women living with HIV, comorbid psychological distress, including depression and anxiety, has been shown to negatively affect daily functioning and quality of life. Suicidal ideation has been reported following diagnosis, with HIV-related stigma perpetuating further social isolation. Many women turn to spiritual and religious practices to cope with psychological distress [24]. In South Africa, women living with HIV experience psychological distress linked to family conflict, violence, and poverty, often relying on emotional support from family members, friends, or religious groups [25]. Qualitative evidence indicates that mental distress significantly influences treatment-seeking decisions and coping mechanisms [26]. Similar findings have been reported in Indonesia, particularly among women caring for children living with HIV [27]. Women living with HIV who experience depression may also use substances as a coping strategy to manage overwhelming emotions and stress and often report social withdrawal, low self-confidence, and a diminished sense of purpose in life [28]. Factors contributing to depression include intimate partner violence, inadequate professional support, and substance use [29].

Individuals employ various coping strategies to manage stress, including problem-focused, emotion-focused, and avoidant coping [30,31]. While some strategies promote psychological well-being, others may exacerbate stress and lead to negative mental health outcomes. Spiritual coping strategies have been shown to positively influence both physical and mental health, with higher spiritual well-being associated with lower levels of depressive symptoms among women living with HIV [32].

In Ethiopia, qualitative studies among women living with HIV and depression indicate that stressful life events significantly affect their well-being. Many women face unemployment, hopelessness, and social isolation and report feeling unaccepted by their communities [33]. Some seek relief through traditional or spiritual healing practices [33]. Although depression is common among women living with HIV, critical aspects such as symptom experiences, perceived causes, aggravating and relieving factors, coping strategies, and major challenge remain underexplored, particularly in Gondar City health facilities.

A qualitative approach is therefore essential to gain an in-depth understanding of these experiences from the perspectives of women living with HIV themselves. Such an approach captures the complexity, context, and personal meaning of depressive symptoms struggles in ways that quantitative methods may not fully reveal [34]. Addressing these gaps is necessary to generate evidence that supports the integration of mental health services into primary healthcare, spiritual care, and HIV service delivery systems.

## Method and materials

The report of the methods section was guided by consolidated criteria for reporting qualitative studies (COREQ) checklist that contains 32 items that supporting the explicit and comprehensive reporting of qualitative studies [35] (S1 File).

### Study design

A phenomenological study design is a qualitative approach that aims to understand people’s lived experience and the meaning they give to them. Instead of focusing on numbers or measurements, it seeks to capture the personal perspectives, emotions, and realities of those experiencing a particular situation. This approach is especially useful for exploring sensitive or complex experiences, such as illness, mental health challenge, or life changes. Researchers typically gather insights through in-depth interviews, conversations, or personal stories, allowing participants to share their experiences in their own words. Phenomenology is often used to inform care, support, or interventions by truly understanding the day-to-day realities of people’s lives for example, learning how women living with HIV experience depression, cope with challenge, and navigate the impact on their daily lives.

### Study setting

The study was carried out in health facilities in Gondar City, north-western Ethiopia, with data collected between August 25 and September 25, 2024. Gondar City is located 728 km from Addis Ababa and has one specialized hospital**,** one primary hospital**,** and eight health centres, with all except Bilajig health center providing ART services. During the study period, 6,042 adult WLHIV were registered for ART follow-up, with most attending the University of Gondar Comprehensive Specialized Referral Hospital (UoGCSRH), the largest facility and the only one offering mental health services at both outpatient and inpatient levels at UoGCSRH.

**Study population and recruitment**: Women aged 18 and above living with HIV who were experiencing depression as determined by as screening too (PHQ-9), attending an ART clinic during the data collection period and who had the possibility of a follow-up interview if necessary were selected. Depression was measured using patient health questionnaires, two weeks before the data collection period. Depression was measured by the nine-item Patient Health Questionnaire (PHQ-9), with each item requiring participants to rate the frequency of depressive symptoms experienced two weeks before the data collection period. The total score ranged from 0 to 27. Those score 10 and above according to PHQ-9, was considered as having depressive symptoms. The severity of depression was assessed using a four-point Likert scale ranging from 0 to 3, with depression levels measured as follows: no depression (0–4 points), mild depression (5–9 points), moderate depression (10 –14), moderately severe depression (15 –19), and severe depression ()20 21–2627 [36]. Purposive sampling technique with criterion sampling technique was used to selected study participants. In phenomenological study design, 5–25 samples are recommended [34]. However, in the current study 16 study participants were included due to saturation. Stopping interviews after data/information saturation is widely accepted as a methodological principle in qualitative research, when no new ideas emerge [37,38]. Women who were too ill, had cognitive or communication difficulties, were under 18, or chose not to provide consent were not included in the study.

### Data collection

The principal investigator (TAZ), a male PhD student with an MSc in Mental Health and extensive experience in qualitative research and mental health studies, personally conducted all a semi-structured in-depth face to face interviews at the health facilities. There was no prior personal relationship between the researcher and the study participants.

Before data collection began, participants were fully informed about the objectives of the study, assured of confidentiality, and provided both verbal and written informed consent. With participants’ permission, all interviews were audio-recorded, taking field notes to accurately and respectfully capture their voices, experiences, and meanings for subsequent transcription and analysis.

Interviews were conducted in a safe, private, and comfortable environment to facilitate open and honest dialogue. All interviews were carried out in Amharic, the local language and lasted between 30 and 70 minutes. Each participant took part in two interview sessions to ensure a deeper and more accurate understanding of their lived experiences. The first session focused on building rapport and trust between the researcher and the participant, while the second session allowed for more in-depth exploration of experiences related to depression.

An interview guide and checklist were developed based on an extensive review of the literature to ensure a logical flow of questions and to minimize ambiguity. To be understandable of the interview guide, simple cultural context words were used. The guide was reviewed and refined in consultation with qualitative research experts, including a Professor and mental health epidemiologist (TA), to enhance clarity and relevance. The component of the interview guide were background information, experience of depressions symptoms, perceived cause of depression, perpetuating and relieving factors, treatment perception, impact of daily life, coping strategies, challenges and probing questions. Prior to the main data collection, the interview guide was piloted with five purposively selected participants from a similar population at a health center, allowing for refinement of questions and interview techniques.

The interviewer’s background and role were openly acknowledged as part of the study. The interviewer was trained and experienced in qualitative and mental health research, which helped create a comfortable and respectful interview environment. Recognizing that personal experiences and assumptions could influence the research process, the interviewer engaged in ongoing self-reflection to remain aware of potential biases. Personal reflections and field notes were used to monitor these influences throughout data collection and analysis. The interviewer’s interest in the topic stemmed from professional and academic curiosity, and care was taken to approach each interview with openness, neutrality, and sensitivity, allowing participants to share their experiences in their own words.

### Data analysis

In this study, an interpretive phenomenology was used to understand, and emphasize the historical and social contexts that shape action when analysing texts [39]. Heidegger explored the nature of being and temporality (an ontological perspective), focusing on human beings as active participants in the world [40]. His concept of the life world suggests that individuals’ realities are inevitably shaped by the environment in which they exist [41]. This study asserts that qualitative research, particularly Interpretative Phenomenological Analysis (IPA), provides a flexible and versatile approach to understanding people’s lived experiences [42]. Data were analysed using Interpretative Phenomenological Analysis (IPA) [43]. Data were prepared for analysis by reviewing audiotapes and field notes, and any transcription clarification or corrections were made. First, the data transcription were made by first author (TAZ), and second, discuss with qualitative research expert (TA). After transcription were done, translation was done by TAZ and TA. Initial coding was done by TAZ and TA. LM, ZAD and TAA reviewed and refined the coding framework, validated the themes, subthemes and ensured consistency in the analysis. All authors contributed to data interpretation, manuscript drafting, and approved the final version. The final transcription was exported to MAXQDA qualitative data management software. The first author (TAZ) read each transcription multiple times and developed a coding scheme with the lead supervisor (TA). Data were coded by two individuals. Data were analysed using Interpretative Phenomenological Analysis, with careful attention to the idiographic and interpretative principles that underpin this approach. The analysis was organised and supported by the five-step qualitative data analysis framework developed by Ritchie and Spencer [44]. These steps include (i) *familiarisation* where data is read and re-read through each transcript, breaking down the data into small chunks of wording, and making comments or labels to the data, (ii) *identification of a thematic framework* by writing down key issues and concepts that recurrently emerged from the data, (iii) *indexing* the *data* by listing the open codes that had been made to the data to identify similar or redundant codes and reduce the long list of open codes to a manageable number, and then a closed coding was carried out to group similar codes under the same themes and sub-themes, (iv) *charting* data through arranging appropriate thematic references in a summary of chart which enabled comparison across interviews and within each interview, and (v) *mapping and interpretation of the data* through which data were examined and interpreted [44]. The used steps assisted the team to manage the qualitative data in a coherent and structured way, and guided the analytic process in a rigorous, transparent and valid way.

The interpretation was then advanced using the interpretive phenomenological analysis (IPA) [45] using inductive IPA. The IPA, emphasizes the historical and social contexts that shape actions when analysing texts [39]. The interpretative IPA, provides a flexible and versatile approach to understand people’s lived experiences [42,43]. Heidegger explores the nature of being and temporality (an ontological perspective), focusing on human beings as active participants in the world [40], and suggests that individuals’ realities are inevitably shaped by the environment in which they exist [41]. Themes were organized into superordinate themes with corresponding subthemes.

### Rigor

Consistent with IPA and to ensure trustworthiness, various qualitative validation strategies are required. To uphold trustworthiness, specific criteria were applied, emphasizing the importance of data integrity in qualitative research [46]. This study adhered to the four trustworthiness criteria established by Lincoln and Guba: credibility, transferability, dependability, and confirmability [47]. Credibility was established by accurately capturing participants’ lived experiences through prolonged engagement, triangulation, and member checking, where participants review and verify interpretations [48,49]. To enhance credibility, member checking was carried out by returning transcripts to 43.8% (seven) of the participants for their review and approval. This approach aligns with qualitative research standards, which recommend validating findings with a representative subset of participants [50]. Dependability was maintained by keeping an audit trail, documenting coding decisions, reflexive notes, and methodological choices to ensure consistency in analysis. Confirmability was achieved through reflexivity, where the researchers continuously engaged in self-examination and critical reflection throughout the research process to reduce researcher bias. Transferability was supported through the use of rich, thick descriptions of the research setting. These strategies collectively enhance the rigor and validity of IPA research [51,52].

### Ethical considerations

This research was conducted in compliance with ethical guideline of the University of Gondar and received approval from the Institutional Review Board of the University of Gondar College of Medicine and Health Sciences (Approval No. 06/02699/8/2023). Additionally, written informed consent, obtained through signature or thumbprint, was secured from all study participants before the investigation commenced. All data collected for the study was handled with the highest level of confidentiality, ensuring that no personal information was disclosed. Participants were explicitly informed that they would not be asked to provide any identifying details, such as their name, last name, address, or phone number. This approach safeguarded their privacy, fostered trust in the research process, and upheld the study’s integrity. Those participants who had suicidal thought were linked to medical and psychiatric clinic with their consent.

## Results

### Demographic characteristics of the study participants

This study included 16 participants aged between 32 and 60 years with 100% response rate. Regarding education, seven participants were unable to read and write, while three had completed high school and the rest were attending elementary school. In terms of marital status, four participants were married, one was single, two were widowed and nine were divorced. Depression was understood through Patient Health Questionnaires, those who scored 10 or more were considered as having depressive symptoms rather than formal diagnostic tools, as the goal was to hear how they lived with and made sense of their emotional struggles. At the time of the interviews, women described different levels of distress, including ongoing sadness, hopelessness, loss of interest, and social withdrawal.

Study participants’ economic situations were also an important part of these experiences, especially for women who were unemployed. Many shared how they struggled to cover daily expenses and relied on family members, small informal jobs, community support, or government assistance to survive and access their medication. These financial challenge were closely tied to their emotional well-being and ability to manage life with HIV. The duration of depression among participants varied from 9 months to 7 years. These were due to depression was assessed by asking participants whether they had experienced depressive symptoms during the previous two weeks. Those who answered “yes” were included in the study, and they were also asked how long they had been living with depression. We included people with a wide range of illness durations because, in qualitative research, diversity of experience helps deepen understanding. Participants who had been depressed for at least 9 months were able to reflect meaningfully on their experiences, while those with longer histories offered valuable insights into what it is like to live with depression over time, including long-term coping and adaptation.

(Table 1).

**Table 1:** Participants’ demographic information in women living with HIV, 2024/2025.

Participants	Age group	Job	Depressive symptoms start
P1	36-40	Daily labourer	9 months
P2	36-40	Daily labourer	7 years
P3	36-40	Cleaner	4 years
P4	31-35	Café waiter	3years
P5	36-40	Unemployed	2 years
P6	46-50	Unemployed	2years
P7	36-40	Housewife	1year
P8	46-50	Waiter	5 years
P9	31-35	Cleaner	1 year
P10	31-35	Cleaner	3 years
P11	33-35	Sex-worker	3 years
P12	40-45	Unemployed	3 years
P13	35-40	Daily labourer	1 year
P14	35-40	Daily labourer	1 year
P15	56-60	Unemployed	4 years
P16	50-56	Daily labourer	6 years

### Thematic framework: Depression in women living with HIV

The study generated seven themes and subthemes lived experience of depression in WLHIV. These are summarised below (Table 2).

**Table 2:** Thematic framework of depressive symptoms in WLHIV in the form of theme and sub-themes 2024/2025.

Theme	Sub-theme
I. Experiences of depressive symptoms	Physical Symptoms Emotional and psychological Symptoms Suicidal thoughts and self-harm Cognitive symptoms Appetite changes Behavioral symptoms Relationship struggles
II. The meaning of depression	A heavy burden Stillness and isolation Emotional turmoil and regret Physical and mental distress Hopelessness and lack of motivation A consuming illness A disruptive force
III. Perceived causes of depression.	Physical Factors Economic Factors Stigma related Factors Social Factors
IV. Perpetuating and relieving factors.	Perpetuating factors(Economic difficulties, social isolation and lack of support, Sleep disturbances and restlessness) Relieving Factors(Social support and positive interactions, Engaging in activities and self-expression)
V. Belief of depression treatment	Holy water and spiritual healing Professional treatment and hope for healing Acknowledgment of uncertainty
VI. The coping strategies	Psychosocial coping strategies Behavioral coping strategies Spiritual coping strategies Modern treatment seeking Emotional coping strategies
VII. Major challenge	Forgetfulness and cognitive difficulties Work loss Loss of confidence Regret Emotional instability Fatigue Neglect of self-care and ART treatment

### Further description of the themes and subthemes is provided below

#### Superordinate theme I: Experience of living with symptoms of depression.

A wide range of crippling symptoms that significantly impacted their physical, emotional, cognitive, and behavioral well-being were reported by study participants who were living with depression. Their first-hand experiences demonstrate how depression affected them in every part of their life, and severely hindered their capacity to function, going far beyond simple melancholy. Majority of the study participants reported feeling of sadness, sleep disturbance and worthlessness. Some of them also reported suicidal ideation and attempt. These depressive symptoms were described in subtheme below.

#### Physical symptoms.

A range of physical symptoms related to depression were reported by the participants. Common symptoms were fatigue, chronic physical weariness, which made simple daily tasks burdensome. Additionally, many reported experiencing pressure-like sensations in various body areas, numbness, dizziness, and body aches.

*“I experience headaches, body aches, and numbness in my legs and waist”* (a 35–40 year old daily labourer*.*

#### Psychological and emotional symptoms.

Problem in sleep patterns was a common and distressing symptom reported by a majority of participants, significantly impacting their overall well-being and ability to function. These disturbances ranged from chronic insomnia, where individuals struggled to fall or stay asleep, due to this, they still feel exhausted and unrefreshed. For some, insomnia was linked to persistent feelings of anxiety, racing thoughts, and emotional distress. Even when they managed to fall asleep, most found themselves waking up frequently throughout the night, experiencing restless and fragmented sleep that left them drained the next day and suicide ideation.

*“At night, I wake up and sit in front of the TV until midnight. I shift from bed to chair, hoping to fall asleep, but it doesn’t help” (a 31-35 year old clear). “On nights when I can’t sleep, thoughts of ending my life creep in” (a 36-40 years old daily labourer).*

Others reported experiencing vivid nightmares or distressing dreams that intensified their emotional suffering. Some participants described waking up feeling panicked, disoriented, or emotionally overwhelmed, making it even more difficult to return to sleep. The anxiety induced by these nightmares often carried over into their waking hours, contributing to a heightened sense of fear and unease. *“I have unsettling dreams of being thrown off a cliff, chased by a man, or eaten by a dog. (….after waking up, it is difficult to sleep again…I fear that occasion will occur again. During the distressing dreams, I felt shortness of breath, palpitation, anxious, restless, shouting…” (a 36–40 years old divorced). Another a 40–45 years old beggar stated that “ As my family told me in the midnight, I tried to open the door to go out and at times even attempted to harm myself, however, I didn’t remember the events.”*

On the other end of the spectrum, some participants experienced hypersomnia, sleeping excessively yet still feeling fatigued and unmotivated. However, despite sleeping for long hours, they reported feeling sluggish, mentally foggy, and physically drained, further exacerbating their depressive symptoms. Due to depression at initial time, there was excessive sleepiness.

*“The most challenging aspect for me is excessive sleepiness. I even fall asleep while making coffee or caring for my child” (a 31-35 year old daily labourer).*

Depression left many participants feeling isolated, disconnected, and without purpose. The recurring themes of regret, sadness, and a lack of hope for the future emphasized their emotional struggles.

*“I feel extremely alone, without support or family, and often cry in silence.” (a 46-50 years old divorced unemployed); “I feel like my life has no purpose, and I rarely think about the future.” (a 36-40 widowed daily labourer)*

#### Suicide ideation and attempt.

For some participants, the overwhelming emotional distress led to thoughts of self-harm or suicide. Many described feeling trapped in their suffering, with some contemplating specific methods of ending their lives. The focus on “hanging” or “drinking poison” suggests that the person may feel suffocated or poisoned by their emotions, which may lead them to see death as a way to escape this pain.

*“I think about hanging myself or drinking poison to end my life and get rest” (a 56-60 year old beggar).* The individual feels burdened by the suffering of the world, potentially indicating feelings of powerlessness or a sense of being trapped by their circumstances. *“I have dark thoughts, I’ve contemplated suicide as a way to escape this despair. At times, I think about ending my life using electric shock or a rope at night, but my husband prevents me” (a 31-35 year old daily labourer).*

#### Cognitive symptoms.

The majority of participants reported significant cognitive challenge, including difficulty with memory, concentrating, and impaired decision-making, which deeply affected their daily lives. Forgetfulness was a persistent struggle, with some participants frequently missing appointments, misplacing important items, or failing to recall conversations. This memory decline created frustration and self-doubt, further exacerbating their emotional distress.

Difficulty concentrating made even routine tasks feel overwhelming, as participants found themselves easily distracted or unable to focus for extended periods. Some described zoning out during conversations, struggling to retain information, or feeling mentally exhausted after simple activities. This lack of focus hindered their ability to perform at work, complete household responsibilities, or engage in social interactions, reinforcing feelings of inadequacy.

Impaired decision-making was another major concern, with participants expressing anxiety over even minor choices. They reported second-guessing themselves, feeling paralyzed by indecision, or making impulsive choices they later regretted.

*“I often forget things and wonder what’s happening to my memory.” (a 41–45 year old beggar);*

#### Perceptual symptoms.

One study participants reported that whenever she saw her child, her child seems a puppy. This may reflect cognitive overload or impaired attention, possibly linked to depression or emotional exhaustion. There was also a perceptual distortion, potentially linked depression.

*“My mind drifts when people talk to me, and I struggle to follow conversations.” (a 31-35 year old cleaner); a 31-35 year old daily labourer stated that “There are times when I don’t even recognize my child as my own even when she was crying; I’ve thought of her as a puppy instead (‘ልጄ እያየኋት እያለቀሰች የዉሻ ቡችላ ነበር የምትመስለኝ፡፡)”*

**Appetite issues:** Underlying emotional or psychological issues are disrupting the study participants’ appetites and eating patterns. Food aversion, inconsistent eating patterns, and appetite loss are all signs of how emotional states like worry, stress, or depression can interfere with fundamental bodily requirements and self-care. Hunger cues may be disregarded, forgotten, or not felt at all in these people due to an emotional detachment from their bodily selves.

*“My appetite is unpredictable; sometimes I eat, but other times I go without food for three days because I forget to eat,” (a 51-55 year old daily labourer)*

#### Behavioral symptoms.

Depression led to withdrawal from social and daily activities, often accompanied by mood swings, irritability, and difficulty managing emotions. *“I stay at home, have lost interest in work, and isolate myself from others.” (a 36–40 year old daily labourer)*

#### Difficulties of relationship.

Women living with depression suffer with emotional struggles within relationships, where feelings of disconnection, regret, invalidation, and mistrust intersect. The themes of loss, alienation, and emotional healing are central to these experiences.

*“I feel unsupported, as my mother and others seem to dismiss my feelings. My struggles with intimacy stem from past betrayal, making it difficult for me to trust my partner.” (a 31-35 year old café waiter)*

### Superordinate theme II: Meaning of depression

Depression is experienced as a deeply personal and overwhelming condition that extends beyond clinical definitions. It is shaped by emotional distress, physical sensations, social withdrawal, and its impact on daily life. Participants described depression as an existential burden, affecting every aspect of their being.

**Heavy burden**: Depression was often described as an unbearable weight that makes functioning difficult. Participants felt emotionally and physically drained, as expressed by a 31–35 year old daily labourer: “*Depression is as if I’m weighed down like a lifeless person.”*

**Isolation:** The majority of the participants linked depression with immobility and social withdrawal. It reflected psychological withdrawal and a need for emotional relief, possibly indicating that the home environment is experienced as stressful or unsupportive. A 36–40 year old daily labourer shared, *“I find myself just sitting in one place,”* while a 50–55 year old daily labourer echoed, *“For me, depression is simply sitting in one place.”*

**Feeling of regret:** Depression led to regret and self-directed frustration and emotional pain sometimes come out as anger toward others. A 40–45 year old beggar described, *“It keeps me quiet, and I find myself regretting the past and lashing out at others.”*

**Sense of physical and mental distress:** Depression was not only an emotional struggle but also a physical one. Participants reported sensations of dizziness, numbness, and exhaustion. Depression can represent emotional heaviness, where the person may not openly express her struggle but feels internally overwhelmed, tired or troubled. A 31–35 year old sex-worker expressed, *“Depression is (ዱካክ(Dukak)): It’s a dirty feeling. It just makes me want to sit down and do nothing.”*

**Lack of motivation and hopelessness:** Depression drained participants’ energy and hope, making it difficult to find motivation. A 35–40 year old daily labourer stated, “*It(depression) demotivates me*,” while a 36–40 year old unemployed added, “*It(depression) makes me hopeless.”*

**Impaired function:** Depression affected basic daily functions, including sleep, communication, and emotional stability. A 31–35 year old cleaner expressed, “It (d*epression) makes hard for me to talk to others, it(depression) disrupts my sleep and often brings me to tears. I was absent from work, and the manager gave me a warning letter. Nowadays, my colleagues and manager have started to understand my mental health condition (depression) and show me some care.”*

### Superordinate theme III: Perceived causes of depression

The study participants reported that physical, economic, psychological, and social factors were possible causes for depression. In the psychological cause, stigma takes the lion’s share. Economic hardship, ART side effects, and a lack of social support further intensified their struggles.

**Physical factors/medical conditions:** The burden of living with HIV and the side effects of an ART played a critical role in participants’ depression. Many reported that their depressive symptoms began after their HIV diagnosis. The physical toll of the virus, coupled with ART side effects such as numbness and emotional instability, heightened their distress.

A 31–35 year old daily labourer directly linked her depression to HIV, stating, *“My depressive symptoms began after I was infected with the virus.”* Similarly, a 36–40 year old daily labourer noted, “*I had never experienced illness before, but after being diagnosed with HIV and starting ART, I developed symptoms of despair and depression.*

**Economic factors:** Financial instability was a major contributor to depression, creating stress and uncertainty in participants’ lives. The inability to provide for their families led to feelings of helplessness. A 41–45 year old beggar, for example, described her distress at not being able to support her children, stating, *“When my children ask me what I can do for them, I have no answer. For instance, they (children) asked me to buy bread, however, I couldn’t buy it. So, I felt stress.”* She also lamented that financial struggles forced her children to drop out of school.

**Stigma related factors:** The psychological impact of stigma and discrimination played a profound role in participants’ depression. The fear of rejection, social exclusion, and being defined by HIV status intensified feelings of isolation.

A 31–35 years old cleaner shared how people identified her by her HIV status rather than her name, stating, “*People refer to me by my virus status instead of my name, so I’ve withdrawn from social activities. It causes me a lot of stress (…).”*

Disclosing HIV status to friends and family, worried that it would lead to insults and rejection. A 31–35 year old daily labourer stated *“Disclosing my HIV status to friends and family has led to insults and rejection. Even without disclosing it, people judge me based on my appearance and suspicions. We used to drink coffee together, but once my neighbors suspected me, they started ignoring me. Now, I spend most of my time alone behind closed doors, crying. It leaves me feeling hopeless, isolated, and in despair.”*

Stigma extended to participants’ family including children. A 40–45-year-old beggar revealed that *“Even though my children can’t attend school now because of financial hardship, back when they (children) did, they were stigmatized. Their classmates said, ‘Don’t greet or play with them, their (children’s) mother has HIV.’ Because of that, my children eventually refused to go to school. It made me feel deeply depressed.”*

HIV has led some study participants to experience signs of premature aging, which has become a source of worry and distress for them. A 31–35 year old sex-worker reported that, *“Having HIV has made me look older. When I compare my photo from before I was diagnosed (HIV) to how I look now, I can see the difference, now I look much older.* She showed the interviewer the old photo and began to cry. *Now I feel sadness, hopelessness, and even fear that I will die soon.”*

A 31–35 year old café waiter feared family rejection, stating, “*If my family found out I had HIV, they would see it as a weakness and insult me. I wouldn’t be able to handle it.”*

**Avoidance and isolation**; to reduce stigma and discrimination, the study participants preferred to avoid and self-isolation. A 31–35 year old married daily labourer described *“****I feel as if my HIV status is written on my forehead...*** *(…) I tried to hide my face to avoid being recognized and stigmatized.”*

**Social factors:** A lack of social support and negative community reactions left many participants worsening their depression. The absence of emotional support from family and friends made it harder to live.

A 36–40 year old cleaner shared that a lack of social support contributed to her divorce, which worsened her depression. “*The lack of social support makes it even harder,”* said a 40–45 year old beggar, while a 31–35 year old cleaner stated, “*The lack of support from the community adds to my worries.”*

### Superordinate theme IV: Perpetuating and relieving factors for depression

Depression among participants is influenced by various external and internal factors. Financial instability, social isolation, and sleep disturbances emerge as major triggers, while spiritual practices, social support, and engaging in activities provide relief.

#### Perpetuating factors.

Economic difficulties contribute significantly to participants’ depressive symptoms. The inability to provide for children leads to deep emotional distress, as expressed by a 40–45 year old beggar stated that *“When my children asked me for bread and I couldn’t provide for them, it deepened my depression and made me wish to harm myself. Financial hardship has been the main factor worsening my condition.”* A 36–40 year old unemployed added *“…even though I live with my husband, who works as a daily laborer, we haven’t been able to support ourselves. Because of this, my beloved son now lives with his grandparents on his father’s side. I often cry, thinking that if I had the money, I could raise my son myself. During these times, my depressive symptoms like hopelessness, fatigue, sadness, frequent crying, regret, suicidal thoughts… become even worse. I believe that if the government provided me with financial support, my depressive symptoms would ease”*

**Social isolation and lack of support:** Lack of social support and social isolation worsen depressive symptoms. Participants report that community rejection, separation from loved ones, and stigma related to their HIV status led to loneliness and emotional distress.

A 31–35 year old café waiter reported that *“Before I contracted HIV, I was healthy and I had weight gain but after the infection, I lost a lot of weight. People around me began to stigmatize me, saying I had HIV because my previous boyfriend died from it. In my experience, especially in Ethiopia, people’s attitudes toward HIV have not changed they continue to judge and isolate. Because of this stigma, I often miss work, and receiving social support has been extremely difficult for me.” Additionally, a 60 year old beggar stated that “Receiving social support has been extremely difficult. Even when I’m unwell, there’s no one to offer me even a glass of water. In those moments, I’m overwhelmed by hopelessness and sometimes feel like ending my life.”*

**Sleep disturbances and restlessness:** Sleep disturbances are another major trigger for worsening depression. Participants report that night-time is particularly difficult, as depressive symptoms become more intense.

A 31–35 year old café waiter reported that *“Before I began experiencing depressive symptoms, I used to sleep well at night and take naps during the day. But now, I can’t nap during the day at all, and at night, I can’t sleep without alcohol. When I compare my past to how I am now, I am constantly in tears. Day by day, my depressive symptoms worsen, mainly due to the ongoing sleeplessness. Even in the mornings, I wake up feeling dizzy and exhausted.”*

#### Relieving factors.

Many participants find comfort and relief through spiritual engagement. Faith provides them with a sense of hope, inner peace, and belonging. *A 56–60 year old beggar stated that “I go to church on weekends to pray and attend worship. Getting advice from the priest helps me feel better.”*

**Social support and positive interactions:** Encouragement and social interaction help counteract isolation and provide emotional relief. A 31–35 year old café waiter states that *“receiving care and attention from others significantly reduces my distress.”*

A 56–60 year old beggar reported that “*When I beg near the church, those who encourage me and give advice on how to cope with this economic hardship make me feel happier and relieve some of my depressive feelings. Social support means more than just money. Some people invite me to different ceremonies and treat me like a human being. That makes me believe I am equal to others…. (Begins to cry).*

**Engaging in activities and self-expression:** Participating in small daily activities helps alleviate some depressive symptoms. Simple routines, such as drinking coffee, provide a calming effect for some participants. Family daily practice provide comfort, stability, or temporary emotional relief from distress. Individuals use ordinary, culturally meaningful practice to manage psychological pain and regain a sense of control and emotional balance. A 40–45 year old beggar shares that *“Drinking coffee helps me feel better.”*

### Superordinate theme IV: Belief of depression treatment

Participants had mixed perceptions of depression treatment, with many relying on spiritual practices such as holy water and repentance, while others considered professional treatment. Some saw a combination of spiritual and medical approaches as viable.

**Holy water and spiritual healing:** Holy water was a common first step toward healing. A 36–40 years old unemployed credited it with past improvements: *“Over time, those problems improved thanks to holy water.”* A 31–35 year old café waiter shared, “*When I was baptized, I felt a sense of relief that lasted for a month. I truly believe that depression is a condition that can be treated.”*

**Professional treatment and hope for healing:** Some participants acknowledged the potential benefits of professional treatment. A 36–40 year old cleaner expressed confidence in improvement with the right support: *“(…) for me with the right professional support, there is potential for improvement of depressive symptoms in the long duration.”* A 31–35 year old daily labourer stated, “*I believe that depression can be self-limiting. Unlike HIV, which has medication, I don’t think depression is a medical illness because it doesn’t have any medicine.”*

**Acknowledgment of uncertainty:** Others expressed uncertainty about the future of their depression. A 36–40 year old housewife said, *“I believe it can get better…, I still don’t have anything that truly addresses the underlying problem.”* A 36–40 year old daily labourer, were uncertain about the future, stating, *“I’ve never thought about the future. Let’s hope so.”* A 36–40 year old daily labourer stated that *“If Jesus Christ can help me improve, then I believe I can help myself too.”*

### Superordinate theme VI: Coping strategies of depression

WLHIV faced emotional distress due to discrimination, stigma, health challenges, and socioeconomic hardships associated with their condition. This emotional distress leads to depression, in turn affects overall health, and quality of life. How they cope with their depression is multifaceted, shaped by their personal experiences, cultural beliefs, social environments, and available support systems.

Coping mechanisms among these women are diverse and can be categorized into psychosocial, behavioral, and spiritual approaches

While some of these coping strategies are adaptive, helping women constructively manage their emotional pain, others may be maladaptive, providing only temporary relief while ultimately exacerbating their distress.

#### Psychosocial coping.

Many participants described self-isolation as a primary way of coping with their depression. Faced with overwhelming emotions, they withdrew from social interactions, preferring to be alone rather than engage with family, friends, or their community. This self-imposed isolation often stemmed from a fear of judgment, stigma, or the belief that others would not understand their struggles.

*“I spend time alone, go outside, sometimes sit in one place for hours because of my depression,”* a 31–35 year old cleaner. *“I tend to stay quiet for a while. I sit at home and often stay in bed”* a 51–55 year old daily labourer.

**Living for others:** Despite experiencing feelings of hopelessness and worthlessness, some participants found motivation to continue living for the sake of their loved ones, especially their children.

*“I have two children, ages 12 and 5, and I live for them. They would be happy just to see their mother, even if I’m not doing much for them” a 36–40 year old cleaner. “I think about wanting to die, but then I wonder who would care for my mom if I were gone” A 31–35 year old café waiter. “I don’t see any value in myself (…). I live for my daughter” A 36–40 year old daily labourer.*

#### Behavioral coping strategies.

**Substance use as a coping mechanism:** Some participants used substances including, alcohol, chat, and coffee, as a way to cope with their depression and find short-term relief from emotional problems. They found that these substances provided a temporary escape, allowing them to feel relaxed, distracted, or even socially engaged. However, this relief was short-lived, as the effects of the substances eventually wore off, often leaving them feeling even more overwhelmed, anxious, or depressed than before.

*A 31–35 year old café waiter stated that “When I drink alcohol, I feel a temporary relief and think I’m having fun. (…) the next morning brings even more overwhelming feelings of depression and heightened anxiety. I find that I either have to drink alcohol to laugh or remain silent (crying)). When I feel overwhelmed, I immediately seek out a friend who drinks and can keep me entertained. Once I have one or two bottles of beer, I feel like I can manage my problems. I’m open to listening to whatever anyone has to say. It helps me fall asleep for a short time, but once it wears off, I wake up again in the middle of the night. Alcohol has become a habit for me to help with sleep, but it only leads to more sleeplessness as soon as it runs out.”* Similarly, a 35 year old sex-worker stated that *“I drink beer and Tela (local alcohol) to help me relax a little. (… which makes me feel sleepy). I chew chat and smoke five sticks cigarettes while drinking beer, which provides a temporary relief. (… my troubles and depressive symptoms will return tomorrow. My living condition with substance is (“****ዉሃ ቅዳ ዉሃ መልስ”) like “water returns to water.”*** This proverb conveys the idea that things often return to their original state or that similar things tend to come together again and again. Some participants used coffee as a form of emotional relief, even though they recognized that it did not ultimately alleviate their depression. A 31–35 year old daily labourer; *“I drink coffee, I’m addicted to it; I feel like I wouldn’t be human if I didn’t have my daily cup of coffee. Sometimes, even after having coffee, my depression doesn’t improve. “*

**Suicidal thoughts:** Many participants grappled with persistent suicidal thoughts as they struggled to cope with the overwhelming emotional pain and deep-seated feelings of worthlessness. Some described moments when they felt completely consumed by despair, believing that their lives no longer held meaning or value. In these darkest times, suicidal ideation became a recurring thought, a perceived escape from their suffering.

*”I sometimes find myself wishing to end my life,” A 56–60 years old beggar. “I sometimes struggle with suicidal thoughts, particularly involving electricity“ a 36-40 years old unemployed. “I wanted to die. The pain inside me is overwhelming, and I feel so lonely there’s no one on my side (crying)” a 31–35 years old daily labourer. “*

#### Listening to music.

**Music as a mental relax:** For some participants, music served as a valuable coping tool, offering a mental escape from overwhelming emotions and providing temporary relief from depression. Listening to music helped shift their focus away from their struggles.

*“I went to my friend’s house to relax and listen to music, which helps me forget about everything,” a 36–40 year old cleaner.*

**Spiritual coping strategies:** Religion played a central role in managing depression for many participants, with practices like prayer, attending church or the mosque, and using holy water providing emotional and spiritual relief. Despite challenge like physical distance from places of worship, faith remained a vital source of support in their coping journey.

*“When I attended church and was baptized, I felt a sense of relief from my depression” A 31–35 year old café waiter. “I feel better when I go to church and spend time getting advice with a priest” A 56–60 year old beggar.* However, some of the study participants began avoiding to attend religious practice after having depression*. “…after having depressive symptoms, I have avoided going to church. This is because life has no meaning (…)” a 50–55 year old daily labourer. “(…I also dislike going to church)” A 36–40 year old daily labourer. “I don’t want to go to church” A 36–40 year old cleaner.*

**Modern treatment seeking:** Some participants recognized the importance of seeking professional mental health support. They consulted healthcare providers when struggling with medication adherence or when their depression became overwhelming.

*“When I take too much medication or forget to take it, I come in for a consultation. However, there is no mental health professional available to treat me,” A 36–40 year old cleaner*.

A 46–50 year old waiter stated *that “even though I tried to get mental health service in the health centre, there is no health professionals that can give mental health service. (They refer me to the hospital, but I couldn’t afford for transport and treatment….)*

#### Emotional coping strategies.

**Crying as an emotional release:** Crying served as a common emotional release for participants experiencing depression, offering temporary relief from their emotional burden. Even when difficult or without a clear cause, crying provided a sense of cleansing, allowing them to feel lighter and momentarily free from their depressive symptoms.

*” My emotions can be intense, and I get angry easily, but crying brings me a sense of relief”” a 51–55 year old daily labourer.*

*“(…) crying, at least, offers me a moment of relief” a 46–50 year old waiter.*

*“(…in tears, and sometimes that brings me a sense of relief),” a 36–40 year old daily labourer. “When I feel depressed, I cry, and it helps me feel a bit lighter,” a 36–40 year old cleaner.*

### Superordinate theme VII: Major challenge of depression in women living with HIV

Depression impacts on women living with HIV, affecting nearly every aspect of their lives, from cognitive function to social relationships, employment, and overall well-being. Depression often led to social withdrawal, making it difficult for women to maintain close connections with family, friends, or romantic partners.

#### Forgetfulness and cognitive difficulties.

Depression severely impairs cognitive function, causing memory lapses, confusion, and difficulty managing daily tasks. Many participants reported forgetting essential activities, such as taking medication or completing simple household chores. Some developed coping mechanisms, like using visual cues to track medication intake. These challenge highlight the profound effect of depression on daily functioning.

**Memory lapses:** Participants frequently forgot small but crucial tasks. *A 46–50 year old waiter shared, “I forget small things, like whether I’ve turned on the kettle or put bread in the oven. “I even left for a neighbor’s house without turning off the oven. It reminded me of times when others shared similar experiences, like causing electrical issues (…)”*

**Disorientation:** Some experienced episodes of confusion and getting lost, as a 31–35 year old daily labourer recalled, “*I sat down, stood up, and forgot what I was about to do.”* A 36–40 year old cleaner struggled with remembering past friends, stating, *“Sometimes, I wonder if I grew up with someone, only to realize I’ve completely forgotten them. Do you think how much the virus affected me? (crying). Because of this I spent more time at home”*

**Work loss:** Depression significantly diminishes motivation and productivity, leading to job loss and financial distress. Sleep disturbances further hinder work engagement, making employment difficult to maintain. *A 36–40 year old daily labourer* noted, “*It’s difficult to stay engaged in work when I feel so unmotivated.”*

**Loss of confidence:** Depression erodes self-esteem, causing social avoidance and isolation. Many participants found it difficult to engage with family and friends. A 36–40 year old daily labourer admitted, *“I tried to go my families’ home, and I remembered that my families might know my HIS status, if so, they could stigmatize me. Moreover, I wanted to visit my family, but my depression held me back.”* A 31–35 year old waiter added, *“I saw myself as lower than the other because I have HIV (….). Even when I speak loudly, I don’t think people hear me.”*

**Regret:** Depression often leads to excessive rumination on past mistakes, fostering a cycle of negative thoughts. *A 56–60 year old beggar* shared, *“I once owned many properties, but I lost everything and had been surviving by washing clothes for others. During that time, I contracted HIV. When I reflect on my situation, I often feel deep regret (…) (…) I constantly live on my past mistakes, but I realize it’s unproductive.”*

#### Emotional symptoms.

Depression leads to emotional disconnection and difficulty expressing feelings, causing frustration in relationships. Women reported experiencing intense frustration, irritability, and emotional outbursts.

A 36–40 year old daily labourer explained, *“When others talk, I feel unhappy but don’t understand why.”* She expressed self-directed anger, stating, “*I know I won’t be as happy as my friends, but I don’t want to be like them either. This makes me angry with myself.”*

#### Physical challenge.

**Fatigue:** Depression causes persistent exhaustion, making even basic tasks feel overwhelming. Participants described feeling physically weak and struggling to complete daily activities. A 36–40 years old daily labourer said, *“The day before, I had agreed to do a day job, but in the morning I felt so fatigued that I couldn’t even answer the phone*. I *feel so tired that I struggle to get out of bed. Some days, it feels like I’m stuck and can’t move.”* A 41–45 years old beggar shared, *“When I felt depressive symptoms, it is difficult to wake up, washing hand, face, plate (…..)I often need to sit down to catch my breath.”*

#### Neglect of self-care and treatment.

Depression significantly disrupts adherence to HIV treatment, making it challenging for study participants to consistently take their medication and attend necessary medical appointments. The overwhelming emotional burden of depression characterized by persistent feelings of hopelessness, fatigue, and cognitive impairment reduces motivation and focus, leading to frequent lapses in treatment routines.

*A 31–35 years old cleaner*, experiences issues such as missed appointments or forgotten medication. *“Depression can affect me by leading to missed medication doses, forgotten appointments, and neglecting invitations or where to put money.”*

*A 36–40 years old daily labourer stated* “*I often forget to take my medication on time and have missed some of my appointments. Because of this, I developed oral thrush. It’s difficult for me to chew or even swallow food and water. I can show you. She then opened her mouth, and the interviewer observed visible signs of oral thrush and referred her to the ART clinic for further care.”*

*“I feel hopeless and don’t want to take my medication. I often miss my appointments, and I find myself thinking that there’s no meaning to life in this world,” (a 41-45 beggar).*

## Discussion

The findings show that women living with HIV experience depression as a complex and deeply personal condition shaped by emotional and social factors. The symptoms such as social withdrawal, hopelessness, frustration, and suicidal thought, reflects not just illness, however, a sense of being overwhelmed and emotionally drained.

Women viewed their depression as arising from the combined burden of illness, emotional pain, stigma, isolation, and financial hardship, which led to exhaustion, despair, and low self-worth. Loneliness and economic struggle were key perpetuates that intensified their distress.

Many understood depression not as a medical condition but as a loss of meaning and hope, shaping uncertainty about treatment effectiveness. Despite this, they sought relief through social support, faith, and professional care in efforts to regain emotional balance.

Participants describe using behavioural, psychological, and spiritual coping strategies to manage distress and regain control. However, their struggle were intensified by regret, unemployment, divorced, memory problems, low confidence, poor ART adherence, and weight loss, reflecting depression as an embodied and deeply personal experience shaping their identified and future.

Depression affected women’s physical, cognitive, emotional, and behavioral wellbeing, with symptoms such as pain, fatigue, sleep problems, forgetfulness, and low self-esteem. They also experienced despair, social withdrawal, loneliness, and suicidal thought or attempts. These findings are consistent with the previous studies showing that WLHIV commonly experience sadness, fear, anxiety and persistent fatigue, often triggered by learning their HIV status [53,54]. Evidence also highlights that sleep disturbances, including insomnia or excessive sleeping, further impair daily functioning and overall well-being [23,27,55–57]. Cognitive symptoms including difficulty concentrating, memory problems, and impaired decision making, affected daily functioning and contributed to feeling of inadequacy. These findings are supported by a evidence that WLHIV with severe depressive symptoms often experience reduce cognitive functioning particularly attention deficits [58].

Women living with HIV perceived depression as a deeply personal burden that affects women’s lives. They perceived depression as making the person demotivated, immobile, isolated, sitting in one place, with feelings of heaviness, dizziness, and numbness. These findings align with previous research showing that depression leads to hopelessness and withdrawal, with individuals perceiving their social world as threating, which further reinforces isolation [53,54].

Depression among participants was shaped by intertwined physical, economic, psychological, social factors, with HIV related stigma as a central sources of distress, making their experiences complex and overwhelming. Many linked their symptoms to the physical burden of HIV and side effects of ART, such as numbness, emotional symptoms, and adherence challenge.

This aligns with evidence that HIV can affect brain function, including serotonin levels, contributing to depression [59].

Economic constraints further affected women’s lives, as financial instability contributed to unemployment, helplessness, and difficulty meeting basic needs. This is consistent with evidence identifying unemployment and low income as key risk factors for depression [60]. Financial hardship can also limit social engagement and increase withdrawal, often driven by stigma or embarrassment, leading to loneliness and worsening depression [56,57].

Psychological factors such as stigma, discrimination, and social exclusion intensified feelings of isolation and worthlessness, leading some women to avoid social interactions due to fear of rejection. This aligns with evidence that internalized HIV stigma is strongly associated with depressive symptoms and poorer psychological adjustment to diagnosis and treatment [61].

Social factors, particularly limited support from family and the community, left many women feeling abandoned and struggling to cope alone. This align with evidence that poor social support contributes to depression among WLHIV by straining relationships, reducing daily functioning, and limiting their ability to meet household responsibilities [56]. In some cases low perceived support can further deepen isolation and intensify depressive symptoms [56].

Depression among participants was driven by financial instability, social isolation, stigma, and sleep disturbances, with intensified emotional distress and feelings of helplessness. Economic hardship, especially difficulty providing for children, and HIV related stigma contributed to loneliness and social withdrawal, while sleep problems worsened mood and exhaustion. These findings are consistent with evidence that poverty, stigma, living alone, and challenge with ART adherence increase the risk and severity of depression among WLHIV [61–64].

Despite these challenge, women used various coping strategies to manage their distress. Spiritual and religious practices provided hope, inner peace, and a sense of belonging, while social support and positive interactions helped reduce isolation. Engaging is small daily activities, such as conversations or drinking coffee, offered temporary emotional relief and a sense of normalcy. Although these strategies did not eliminate depression, they helped women cope, and existing evidence shows that social and professional support can further improve mental well-being [56,64].

Women living with HIV and depression used diverse coping strategies involving psychosocial, behavioral and spiritual approaches. Psychosocial coping included seeking support from family and friends, although some women withdrew due to stigma. Evidence shows that while social support can ease distress, avoidant coping and isolation are linked to poorer mental health outcomes, whereas positive coping and spirituality promote better adjustment [25,65].

Behavioral coping ranged from adaptive activities, such as listening to music, to maladaptive behaviors like substance use, which provided temporary relief but worsened distress over time. This is supported by findings indicating that women with depression often experience depressive symptoms that act as a catalyst for increased substance use, as they turn to substances as a coping mechanism for their depression [29]. Spiritual practices, including prayer, church attendance, and holy water use, offered comfort and a sense of peace and were associated with reduced depressive symptoms. However, some women avoided these practices or experienced severe distress, including suicidal thoughts, highlighting the need for professional mental health support. Overall, while strategies like crying and music offered short-term relief [66], integrated psychosocial and clinical care remains essential [67]. The reason could be listening to music has been found to reduce stress by affecting the hypothalamic-pituitary-adrenal axis and the autonomic nervous system, which lowers cortisol levels and reduces heart rate and blood pressure [66]. In terms of social support, women often turn to health professionals for help with depression [29].

Depression among WLHIV profoundly affects cognitive function, employment, relationships, and overall well-being. Many face unemployment and social disengagement, leading to isolation and limited interaction with others. These findings align with evidence that women often feel rejected by their communities and hopeless about the future due to living with a chronic illness, sometimes turning to traditional treatments for relief [33].

Confusion, forgetfulness and impaired decision making are common effects of depression that interfere with daily functioning. Other findings in chronic illness revealed that cognitive difficulties such as forgetfulness, confusion, and impaired decision-making hinder their ability to manage daily tasks, medication adherence, and work responsibilities [68].

Depression is associated with unemployment and financial hardship, as reduced motivationion and low energy can limit work participation. Stigma, self-isolation, and social withdrawal further worsen functional impairment. It also contributes to fatigue, emotional symptoms, and poor self-care, including missed medication doses and medical appointments. Studies show that individuals with depression often experience impairments in executive function, memory, and attention, which can hinder daily routines, treatment adherence, and work performance [69]. As the result, missed appointment and medication non-adherence are common, particularly among those managing chronic conditions [70]. Depression is also linked to reduce workplace productivity and higher absenteeism [71]. In women, loss of interest in activities may further contribute to unemployment and financial strain [72].

Social withdrawal is a feature of depression, as women may feel unworthy and experience stigma. Research indicates that depression is associated with social disconnection, which can further worsen symptoms [73]. Feeling of being a burden may also contribute to strained or broken relationships [74]. Depression is additionally characterized by emotional symptoms, apathy, and fatigue, which can lead to poor self-care, including inadequate hygiene, reduced engagement in daily activities, and medication non-adherence. Consistent with other findings, such as poor self-care behaviors are associated with poorer health outcomes and increase hospitalization [75].

### Strength and limitation

This study explored depression among WLHIV by focusing on its emotional, social, psychological, and economic dimensions, alongside biological, cognitive, and spiritual influences. It highlights coping strategies, social determinants such as economic hardship and psychosocial stressors, and women’s perceptions of treatment, including reliance on traditional healing and ambivalence toward formal care. The findings also showed how depression affects cognition, employment, relationships, and ART adherence, offering context-specific insights into lived experiences of illness and coping.

Methodologically, the study contribute by applying IPA in this population, providing in-depth understanding of subjective lived experience of depression among WLHIV. However, the study is limited by interpretive nature of IPA, where complete bracketing is not fully achievable, despite efforts at reflexivity to reduce bias.

Additionally, the findings are not generalizable beyond the study participants. Cultural bias in treatment perceptions is another limitation. The study also reflects only women living with HIV, primarily from lower socio-economic backgrounds, which may limit the transferability of the findings to women from more affluent families or different social settings.

## Conclusion and recommendation

Depression among WLHIV poses significant challenge, affecting cognitive function, employment, social connections, and self-care. It also contributes to poor adherence to antiretroviral therapy, weakened immune function, and increased vulnerability to infection. Addressing depression is crucial for ensuring treatment consistency, emotional well-being, and improved health outcomes. A comprehensive approach, including psychological, spiritual, economic, social, and community support, is essential to breaking this cycle and fostering resilience among WLHIV.

To effectively address depression among WLHIV, mental health services have been integrated into HIV care through routine depression screening and psychological interventions. Integrating spirituality with psychological support fosters holistic healing by combining faith-based coping with professional care. Beyond mental health, economic and social empowerment programs such as mental health awareness, sources of treatment, income-generating skill training, financial support, and community-based initiatives are vital for stability and inclusion.

## Supporting information

S1 File COREQ (COnsolidated criteria for REporting Qualitative research) Checklist. (PDF)
